# The diagnostic value of n-terminal probrain natriuretic peptides to differentiate neonatal pneumoniae and transient tachypnea of the newborn

**DOI:** 10.55730/1300-0144.5608

**Published:** 2023-02-04

**Authors:** Zeynep ARSLAN, Serdar ALAN, Didem ALİEFENDİOĞLU

**Affiliations:** 1 Department of Pediatrics, Faculty of Medicine, Kırıkkale University, Kırıkkale, Turkey; 2 Division of Neonatology, Department of Pediatrics, Faculty of Medicine, Kırıkkale University, Kırıkkale, Turkey

**Keywords:** N-terminal probrain natriuretic peptides, transient tachypnea of the newborn, neonatal pneumonia, lung ultrasound, newborn

## Abstract

**Background/aim:**

The primary objective of the study was to determine the diagnostic value of serum N-terminal probrain natriuretic peptide (NT-proBNP) levels to differentiate neonatal pneumonia (NP) and transient tachypnea of the newborn (TTN). The secondary objective was to investigate the prognostic role of NT-proBNP levels in neonates with severe respiratory distress (RD).

**Materials and methods:**

A prospective, observational, single-blinded study involving 58 late preterm and term newborns who were diagnosed with TTN or NP was conducted between June 2020 and June 2021 at a level-3 neonatal intensive care unit in Kırıkkale University Faculty of Medicine. TTN and NP groups were compared for serum NT-proBNP levels measured at the 1^st^ and 24^th^ hours of life. Optimal cut-off NT-proBNP value was determined by Youden index to predict the diagnosis of NP. Lung ultrasound was used to support the diagnosis of TTN and NP. In addition, lung ultrasound score (LUS) was used to determine severe RD.

**Results:**

The median of NT-proBNP level was significantly higher at the 24^th^ hour of life in the NP group than in the TTN group, respectively 7263.5 pg/mL (1643–35,000) and 3308 pg/mL (69–19,746), p = 0.004. At a cut-off value of 5515.5 pg/mL, NT-proBNP had a sensitivity of 75% and specificity of 73.8% to predict NP [AUC= 0.749 (95% CI: 0.602–0.895; p = 0.004)]. The study population was divided into two groups as high score group (n: 23, LUS ≥ 7) and low score group (n: 35, LUS < 7) according to the LUS at the 6th hour of life. NT-proBNP values at 24th hour of life were 6320 pg/mL (69–35,000) in high score group and 3500 pg/mL (570–15,948) in low score group, p = 0.044. Duration of oxygen support (p = 0.006), noninvasive ventilation (p = 0.008) and NICU stay (p = 0.004) were higher in high-score group.

**Conclusion:**

NT-proBNP values at 24^th^ hour of life can be used as a relatively early predictor in the differentiation between NP and TTN in late preterm and term neonates. In addition, elevated NT-proBNP values are related to the higher LUS which reflects the severity of RD regardless of diagnosis.

## 1. Introduction

The most known cause of neonatal intensive care unit (NICU) admission is respiratory distress (RD) [1]. The commonest pulmonary causes are respiratory distress syndrome (RDS) for preterm infants and transient tachypnea of the newborn (TTN) for early term and late preterm infants [1–3]. TTN typically appears within the first few hours after birth and is generally benign and self-limited [4]. Neonatal pneumonia (NP) is another common problem for newborn infants who were admitted to NICU with signs of RD [5]. Differential diagnosis of NP from RDS and TTN is very difficult, especially with the respiratory symptoms and chest X-ray in the first day of the life [2]. The period with the highest risk of death from pneumonia in childhood is the neonatal period, especially in the low-income countries [6]. According to autopsy findings, NP was diagnosed in 20%–32% of newborn infants who subsequently died and early onset pneumonia was described in 10%–38% of stillborn at autopsy [7]. It is very crucial to differentiate NP from TTN for initiating early empirical antibiotics. On the other hand, if we can differentiate TTN from NP, we may prevent unnecessary antibiotic usage. Recently, lung ultrasound has been known to have a higher diagnostic accuracy than chest-X-ray in neonatal RD. In addition, lung ultrasound has been used for the determination of severity of RD and prognosis of pulmonary disease in the newborns [8–10].

N-terminal pro-B-type natriuretic peptide (NT-proBNP) is a 32-amino acid polypeptide which is excreted by ventricular myocytes as a response to volume overload [11]. NT-proBNP is a very beneficial test for the differentiation of congestive heart disease from pulmonary disease in neonatal, pediatric, and adult population [12]. Furthermore, higher plasma NT-proBNP measurements were found in newborns with RD and had a correlation with the severity of RD [13].

The primary objective of this study was to investigate whether serum NT-proBNP levels were useful in differentiating NP and TTN in the newborn. The secondary objective was to investigate the prognostic role of NT-proBNP values in neonates with severe RD findings regardless of diagnosis according to the lung ultrasound score (LUS).

## 2. Materials and methods

### 2.1. Study design and patient selection

This was a prospective, observational, single-blinded study carried out from June 2020 to June 2021 at a level-3 NICU. The study protocol was approved by the Noninterventional Clinical Researches Ethics Committee of Kırıkkale University Faculty of Medicine (2020, decision number: 16/01); written consent was obtained from the parents. The authors have confirmed in writing that they have complied with the World Medical Association Declaration of Helsinki regarding ethical conduct of research involving human subjects and/or animals.

The following were included in the trial: (1) infants who were born between 34^0/7^ and 41^6/7^ weeks of gestation; (2) infants diagnosed with TTN or NP. Patients with hypoxic ischemic encephalopathy, cardiac failure, critical congenital heart defects, congenital anomaly, patent ductus arteriosus (PDA), other causes of RD except TTN or NP (such as RDS, pulmonary hypoplasia, diaphragmatic hernia, and air leakage syndromes of newborn) and patients without parental consent were excluded.

### 2.2. Data collection

Serum NT-proBNP levels were measured at 1st and 24th hours postnatally in all study infants. After admission, postnatal 1^st^ and 24^th^ hours are the routine blood collection times in our NICU for the clinical management of the newborn. Therefore, venipuncture was not performed exclusively for the purpose of this study. About 2 mL of peripheral venous blood taken for routine examinations was separated and added into the serum separator gel tube for NT-proBNP measurement. Blood samples were sent directly to the laboratory within a few minutes. NT-proBNP levels were determined by fluorescent immunoassay (Roche^®^ cobas e 411 analyzer, Indianapolis, USA) with values between 5 pg/mL and 35,000 pg/mL.

Demographic data such as sex, gestational age (GA), birth weight (BW), mode of delivery, APGAR scores at 1st and 5th minutes after delivery, maternal age, history of maternal urinary tract infection, preterm rupture of membrane (PROM), chorioamnionitis, fever, gestational diabetes mellitus (GDM), preeclampsia, and thyroid disease were recorded. The need for positive pressure ventilation (PPV) of the newborn infant after delivery was recorded. In addition, clinical data such as blood pressure, heart rate, respiratory rate, pulse oximetry values, and routinely laboratory values such as hemoglobin (Hb), hematocrit (Htc), white blood cells (WBC), platelets (complete blood count parameters analyzed by Mindray^®^ BC-6800, Shenzhen, China), procalcitonin (Roche^®^ cobas e 411 analyzer, Indianapolis, USA), C-reactive protein (CRP) (Roche^®^ cobas 702 analyzer, Mannheim, Germany), pH, pCO2, HCO3 (blood gas parameters analyzed by Rapidlab^®^ 1200 Systems, Siemens, Chamberley, UK) were recorded at 1st and 24th hours of life. The severity of RD was assessed using Silverman Anderson score at 6th hour of life [14]. Respiratory support data including duration of oxygen support, noninvasive ventilation, need for invasive mechanical ventilation, and also antibiotic usage and duration of NICU stay were recorded.

Chest X-ray (Shimadzu Mobile Art Evolution 32 TC^®^, Shimadzu Corp, Kyoto, Japan) and lung ultrasound (Mindray^®^ M7, Shenzhen, China) were routinely performed for the study population. Lung ultrasound was performed by Z.A. and S.A. who are experienced in neonatal lung ultrasonography. Echocardiography was performed by a pediatric cardiologist using a Vivid 7 Pro (GE Healthcare^®^, Salt Lake City, UT, USA) for those with suspected congenital heart disease and those with symptoms and signs of heart failure or PDA.

### 2.3. Determination of TTN and NP groups

Transient tachypnea of the newborn or NP was diagnosed and treated by an experienced neonatologist who was blinded to NT-proBNP values. Diagnosis of TTN and NP were based on the following criteria and confirmed at the 72^nd^ hour of life.

Transient tachypnea of the newborn was diagnosed based on the following signs and findings: (a) tachypnea with higher than 60 breaths/min, nasal flaring, retractions, and grunting; (b) chest X-ray findings: evident vascular markings, apparent pleural fissures, hilar congestion, signs of air trapping; (c) lung ultrasound findings: pulmonary edema, double lung point, white lung, and compact B-line (d) exclusion of other causes of RD related to the lung (such as RDS, meconium aspiration, and air leak syndromes) and nonrespiratory disorders likely to cause RD (hypocalcemia, hypoglycemia, and polycythemia); (e) improvement of RD signs up to the postnatal 72^nd^ hour [15,16].

Neonatal pneumonia was diagnosed based on the following clinical and laboratory findings: (a) signs and symptoms of RD that did not improve after 72 h of life; (b) higher acute phase reactants; (c) chest X-ray findings: atelectasis, pleural effusion, infiltration, pneumotocele, alveolar densities, etc.; (d) lung ultrasound findings: large areas of hepatizations, irregular margins, pleural line abnormalities, etc. [17].

### 2.4. Determination of RD severity

The lung ultrasound examinations were performed (Mindray^®^ M7, Shenzhen, China) at the bedside at the 6th hour of life and between 24 and 48 h of hospitalization. Each lung was divided into three separate areas; upper anterior, lower anterior and lateral, and examined by a linear probe, through both transvers and longitudinal scans. Findings were obtained in each of three areas of lung, bilaterally. The ‘lateral lung area’ was between anterior axillary line and posterior axillary line. Above the line between the nipples and anterior axillary and parasternal lines was defined as the ‘upper anterior area’. Below the line between nipples was defined as the ‘lower anterior area’. To calculate lung ultrasound score (LUS), a 0–3 score was given for each lung area. Thus, lung ultrasound scores were calculated between 0 and 18 as a sum of both lung areas. Score 0: Presence of merely A-lines; Score 1: Presence of at least 3 B-lines; Score 2: Presence of crowded and coalescent B-lines, absence of A-lines; Score 3: Presence of consolidation [18].

### 2.5. Statistical analysis

SPSS v. 23 for Windows (IBM Corporation Software Group, USA) was used for statistical analysis; a p-value < 0.05 was considered statistically significant. Categorical variables were described as percentage and numbers, and continuous variables as mean ± standard deviation (SD) or median (25p–75p). The distribution of variables was evaluated by visual (histogram graphics) and analytical (the Kolmogorov–Smirnov test) methods. Pearson’s chi-square test was used to compare categorical variables between independent groups. If more than %20 of expected values was <5 or observed value was <2, Fisher’s exact test was used. Two independent groups with normal distribution pattern were compared using Student’s t-test. If these groups were not distributed normally, the Mann–Whitney U Test was used. Pearson’s correlation coefficient was used for jointly normally distributed data that follow a bivariate normal distribution. Spearman’s correlation analysis was used as a measure of a monotonic association. Correlation coefficients (r) 0.0–0.19 were rated as follows: “too weak”, 0.20–0.39; “weak”, 0.40–0.59; “moderately strong”, 0.60–0.79; “strong”, 0.80–1.00. To predict the diagnosis, ROC (receiver operator characteristics curve) with Youden index was used.

## 3. Results

**Figure 1:**
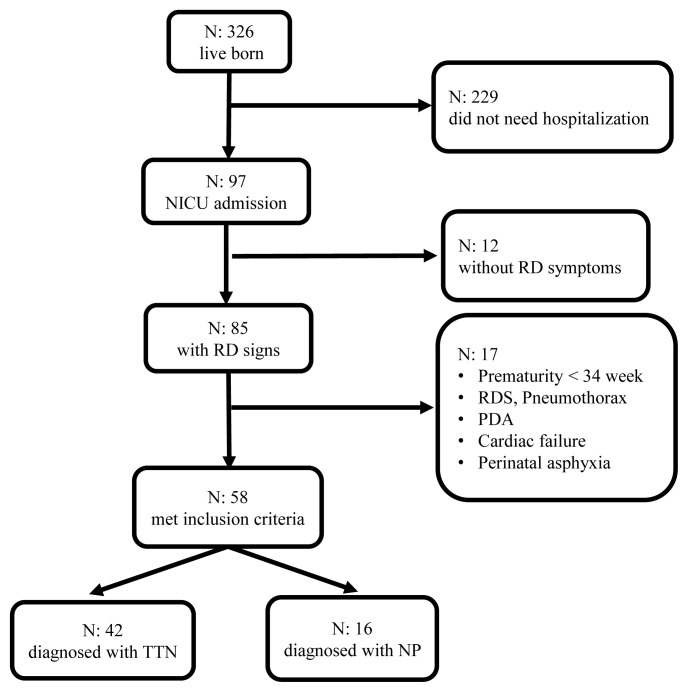
Flow diagram of the study population and diagnostic distribution of the newborns with RD. (NICU: Neonatal intensive care unit; RD: Respiratory distress; RDS: Respiratory distress syndrome; PDA: Patent ductus arteriosus; TTN: Transient tachypnea of the newborn; NP: Neonatal pneumonia).

There were 329 live births in our hospital, 97 of which were admitted to the NICU during the study period. Eighty-five of the 97 hospitalized infants had signs of RD; 58 of them who met inclusion criteria were enrolled. They were divided into two groups by an experienced neonatologist; 42 (72.5%) of the infants were diagnosed with TTN and 16 (27.5%) with NP according to the available criteria (Figure 1). Median GA was 35^0/7^ (34^0/7^–41^6/7^) weeks, and mean of BW was 2589 ± 495 g for infants who were enrolled.

Among all infants included in the study, 15 (25.9%) were term and 43 (74.1%) were late preterm. There was no statistically significant difference in terms of the demographic, maternal, and perinatal data between the groups, except for the male sex, which was significantly more frequent in the TTN group (p = 0.028) (Table 1). The antibiotic therapy rate (p < 0.001), duration of oxygen support (p < 0.001), duration of noninvasive ventilation (p < 0.001), invasive mechanical ventilation rate (p = 0.018), and NICU stay (p < 0.001) were significantly higher in the NP group. There was no significant difference for PPV rate in the delivery room between the groups (Table 2). Platelet count was significantly lower in the NP group (p = 0.032). However, there was no patient with thrombocytopenia in the whole study group. The lowest platelet count was found 152.000 /mm^3^ in the study group. Procalcitonin level was significantly higher in the NP group at the 24^th^ hour of life (p = 0.004) (Table 3).

**Figure 2:**
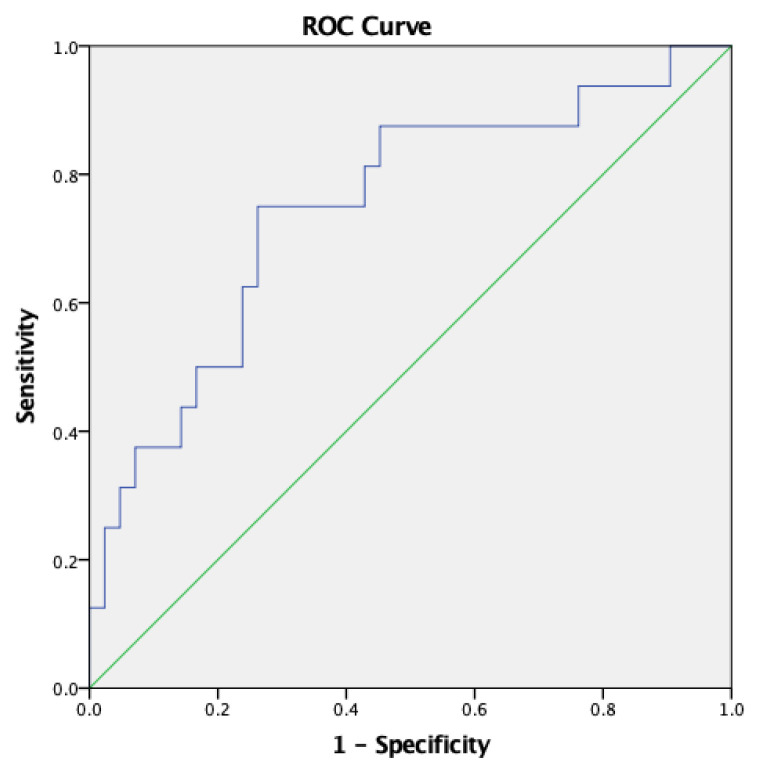
ROC curve for NT-proBNP value to predict the diagnosis of NP at the 24^th^ hour of life.

The median NT-proBNP level tripled at the 24^th^ hour of life and was significantly higher in the NP group [3308 pg/mL (69–19,746) vs 7263.5 pg/mL (1643–35,000), p = 0.004] (Table 3). The optimal NT-proBNP cut-off value was found ≥5515.5 pg/mL by the Youden index to predict NP [AUC = 0.749 (95% CI: 0.602–0.895; p = 0.004)] with a sensitivity of 75% and specificity of 73.8% (Figure 2).

**Figure 3:**
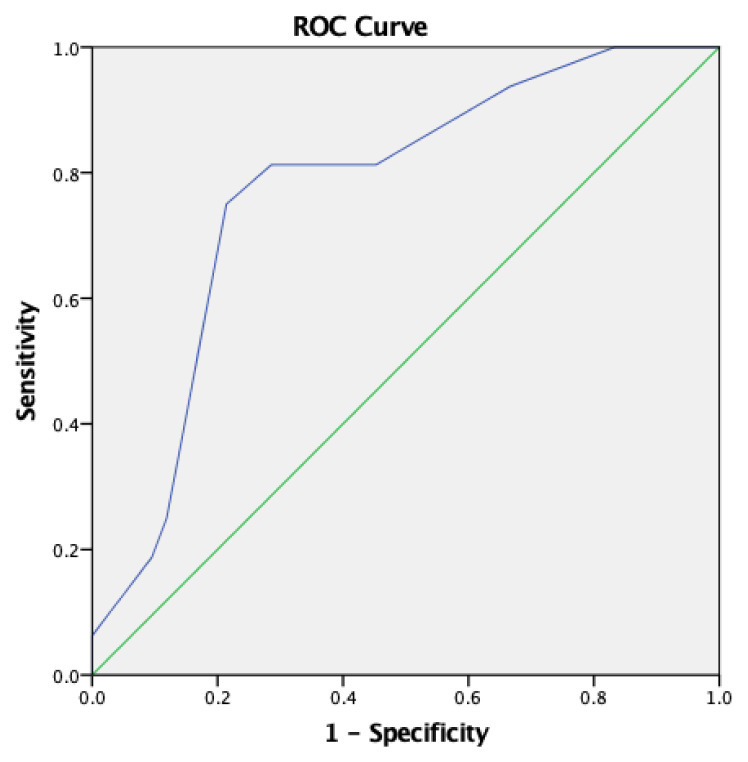
ROC curve for LUS to predict the diagnosis of NP at the 6^th^ hour of life.

The median LUS was significantly higher in the NP group than the TTN group at the 6th hour of life [7 (3–13) vs 4 (1–12), p = 0.001]. The median LUS was significantly higher in the NP group [5.5 (1–12) vs 1 (0–11), p = 0.001] between the 24th and 48th hours of life. The optimal cut-off value for LUS at 6^th^ hour of life was found 6.5 by the Youden index. The ROC analysis for LUS yielded an AUC of 0.772 (95% Cl: 0.640–0.903; p = 0.001). A LUS value greater than 6.5 had a sensitivity of 75% and a specificity of 78.6% for the prediction of NP at 6^th^ hour of life (Figure 3).

When the patients were divided into two groups as a high-score group (LUS ≥ 7 at the 6^th^ hour of life) and a low-score group (LUS < 7 at the 6^th^ hour of life) regardless of the diagnosis, the median NT-proBNP value was significantly higher in the high-score group than the low-score group at 24^th^ hour of life (Table 4). Duration of oxygen support (p = 0.006), noninvasive ventilation (p = 0.008) and NICU stay (p = 0.004) were significantly higher in high-score group (Table 4).

## 4. Discussion

We have shown that late preterm and term newborns with NP had significantly higher NT-proBNP levels than the TTN group at the 24^th^ hour of life. In addition, if the infants had a higher LUS regardless of whether the cause was NP or TTN, they also had significantly higher NT-proBNP levels at the 24^th^ hour of life. However, there was no significant difference in terms of NT-proBNP values between the groups within the first hour of NICU admission.

Pro-B-type natriuretic peptide (proBNP), which consisted of 108 amino acids, is stored in the secretory granules in the atria and ventricles as a precursor form of BNP. The proBNP peaks in the blood within 60 min in response to myocyte stretch due to ventricular volume expansion and pressure overload. The proBNP transforms into BNP, which is a biologically active form of 32 peptides, and NT-proBNP which is a biologically inert form of 76 peptides [19–22]. The plasma concentration of NT-proBNP is higher than BNP concentration due to the lower clearance rate from bloodstream than BNP [22].

Mir et al. [19] described that the mean plasma NT-proBNP level at the 24^th^ hour of life was higher than that on the day of delivery; after this marked increase, NT-proBNP levels decreased steadily and became stable at the postnatal 3rd day in healthy newborn infants. The reason for the high levels of NT-proBNP in the first days might be the adaptation of the circulatory and respiratory systems during the transition period from fetal to neonatal life [13]. According to a more recently published metaanalysis, the mean NT-proBNP values during the neonatal period were found 492 pg/mL and 1341 pg/mL in the umbilical cord blood and serum samples, respectively [20]. Recently, Fritz et al. [23] reported that there was a significant difference in NT-proBNP values according to GA in preterm infants. NT-proBNP values of the preterm infants who were born at <28 weeks of gestation were significantly higher compared with the values of the infants who was born between 28 and 31 weeks of gestation [23]. NT-proBNP levels in the present study were found to be higher than those of the healthy term babies in the literature mentioned above. In addition, there was no data in the literature about the relation with GA and NT-proBNP values for the late preterm and term infants.

There is a lack of data about the relationship between RD and serum levels of NT-proBNP especially in late term and term infants [12,13,24]. Markovic-Sovtic et al. [13] found that term newborns with RD had significantly higher NT-proBNP levels compared with healthy ones, regardless of whether the cause was cardiac or pulmonary disease. However, they suggested that NT-proBNP was not a successful marker in distinguishing the cause of RD (congenital heart disease vs pulmonary disease) in the term newborn babies [13]. On the other hand, Ahmed et al. [12] found that plasma NT-proBNP could be used to differentiate whether RD was of cardiac or pulmonary origin in neonates. In addition, in the study of Cohen et al. [25] in infants with respiratory distress aged 1–36 months, they found that infants with RD due to heart failure had significantly higher NT-proBNP values than those with RD due to pulmonary disease. In the present study, late preterm and term infants with cardiac problems were excluded from the study in order to obtain clear data in differentiation between the TTN and NP groups.

Reel et al. [26] reported in a pilot study that the reason for the increase in NT-proBNP in children with acute lung injury and acute respiratory distress syndrome might be ventricular volume and pressure increase resulting from altered cardiopulmonary interaction. Aydemir et al. [24] indicated that late preterm and term infants with TTN had significantly higher NT-proBNP levels than healthy newborns at 6^th^, 24^th^, 72^th^, and 120^nd^ hours of life. In addition, they suggested that NT-proBNP was a valuable biomarker in evaluating the severity of lung disease because higher duration of tachypnea and need for respiratory support were related to higher NT-proBNP in infants with TTN [24]. In the present study, the duration of oxygen support, noninvasive ventilation, and NICU stay were found significantly higher regardless of the diagnosis (TTN or NP) in the high-score group. In light of these data, high NT-proBNP levels may be correlated with longer respiratory support and hospitalization regardless of the diagnosis (TTN or NP) in late preterm and term infants with RD.

Demographic data of the present study were comparable between NP and TTN groups except for the male ratio which was found to be significantly higher in the TTN group. Nir et al. [27] reported that NT-proBNP levels did not differ between the boys and girls except the 10–14 years age group. In addition, it was already known that male sex is a risk factor for TTN [28]. Seong et al. [29] suggested that the mode of delivery did not lead to significant NT-proBNP level differences in the umbilical cord samples. In the present study, the majority of patients in both groups (90% in the TTN group, 93% in the NP group) were born by cesarean section.

Interestingly, although none of the patients had thrombocytopenia, the platelet count was significantly lower in the NP group in the present study. Although studies show that thrombocytopenia is significantly associated with infectious processes such as sepsis, it is not known whether there is a relationship between lower platelet counts without thrombocytopenia and NP [30].

In a retrospective study by Liang et al. [31], it was reported that procalcitonin levels were significantly higher in infants with NP than in healthy controls. Similarly, we found that procalcitonin values were significantly higher in the NP group. In addition, Bozkaya et al. [32] described that procalcitonin was significantly higher in newborns with congenital pneumonia than infants with TTN or RDS; they also suggested that procalcitonin was an important marker for early diagnosis of congenital pneumonia.

Lung ultrasound was used to support other criteria in the differential diagnosis of TTN and NP. In addition, LUS was used for the determination of the severity of RD regardless of diagnosis in the present study. Recently, Gunes et al. [10] suggested that LUS could be used successfully to predict NIV failure in newborns with TTN or NP. They found that the sensitivity and specificity of LUS to predict NIV failure in newborns differ according to the underlying disorders, and both were higher in neonates with TTN than those with NP. Our study was not related to the role of LUS to predict NIV failure in infants with TTN or NP. However, invasive mechanical ventilation was needed in 4 infants in the high-score group (17%) and only 1 (2.9%) infant in the low-score group. It was not found statistically significant because of the low number of cases in need of mechanical ventilation in the present study. In addition, we found that elevated NT-proBNP values are related to the higher LUS regardless of the diagnosis. Gunes et al. [10] found that higher LUS were found to be related with longer oxygen, NIV, invasive MV durations, and higher rates of antibiotic need in the newborns with RD. Similarly, in the present study, the duration of oxygen support, noninvasive ventilation, and NICU stay were significantly higher in the high-score group.

The present study has several limitations. First of all, the study was a single-center one and conducted with a small number of cases. The second limitation was the low number of infants older than 37 weeks of gestation. The third limitation was that we did not follow up the NT-proBNP levels in infants after the 24^th^ hour postnatal age due to the less invasive care policy in the NICU.

NT-proBNP values at 24^th^ hour of life can be used as a relatively early predictor in the differentiation between NP and TTN in late premature and term neonates. Elevated NT-proBNP values are related to the higher LUS which reflects the severity of RD.

## Figures and Tables

**Table 1:** Comparison of demographic, maternal, and perinatal data between TTN and NP groups.

	TTN (n = 42)	NP (n = 16)	p
**Male, n (%)**	29 (69)	6 (37)	**0.028** 1
**GA, weeks (median (IQR))**	35.5 (34–41)	35 (34–39)	0.297^2^
**Term infants, n (%)**	12 (28)	3 (18)	0.445^3^
**BW, g (mean ± SD)**	2634 ± 498	2472 ± 484	0.270^4^
**C/S, n (%)**	38 (90)	15 (93)	0.691^3^
**Maternal age (years) (mean ± SD)**	29.2 ± 5.3	26.8 ± 3.7	0.102^4^
**Maternal UTI, n (%)**	15 (35)	8 (50)	0.320^1^
**PROM, n (%)**	3 (7)	0 (0)	0.272^3^
**Chorioamnionitis, n (%)**	1 (2)	0 (0)	0.534^3^
**Maternal fever, n (%)**	2 (4)	0 (0)	0.374^3^
**Maternal GDM, n (%)**	4 (9)	0 (0)	0.201^3^
**Maternal thyroid disease, n (%)**	5 (11)	0 (0)	0.149^3^
**Maternal preeclampsia**	4 (9)	3 (18)	0.335^3^
**APGAR (1^st^** **minute), median (IQR)**	8 (5–9)	7 (6–9)	0.802^4^
**APGAR (5^th^** **minute), median (IQR)**	9 (7–10)	9 (7–10)	0.398^4^

1 Pearson chi-square test,

2 Mann–Whitney U test,

3 Fisher’s exact test,

4 Student’s *t*-test.

GA: Gestational age; BW: Birth weight; C/S: Cesarean section; UTI: Urinary tract infection; PROM: Preterm rupture of membranes; GDM: Gestational diabetes mellitus.

**Table 2:** Comparison of the antibiotics, respiratory support data and NICU duration between the study groups.

	TTN (n = 42)	NP (n = 16)	p
**Antibiotics, n (%)**	20 (47)	16 (100)	**0.000** 3
**PPV in the DR, n (%)**	8 (19)	4 (25)	0.720^3^
**Oxygen duration (hours)**	12 (6–96)	120 (9–168)	**0.000** 2
**NIV duration (hours)**	11 (4–96)	84 (9–144)	**0.000** 2
**Mechanical ventilation, n (%)**	1 (2)	4 (25)	**0.018** 3
**NICU stay (days)**	4 (2–13)	9 (7–96)	**0.000** 2

1 Pearson chi-square test,

2 Mann–Whitney U test,

3 Fisher’s exact test.

TTN: Transient tachypnea of the newborn; NP: Neonatal pneumonia; PPV: Positive pressure ventilation; DR: Delivery room; NIV: Noninvasive ventilation; NICU: Neonatal intensive care unit.

**Table 3:** Comparison of vital signs and laboratory results at the 1st and 24th hours of hospitalization between TTN and NP groups.

	TTN (n = 42)	NP (n = 16)	p
**1^st^** **hour of hospitalization**			
**SBP (mm Hg), median (IQR)**	65 (56–88)	63 (47–75)	0.381^4^
**DBP (mm Hg), median (IQR)**	31 (25–52)	32 (22–43)	0.595^4^
**Heart rate, median (IQR)**	149 (102–170)	151 (99–170)	0.882^4^
**Respiratory rate, median (IQR)**	59 (45–68)	58 (48–76)	0.969^4^
Silverman score, median (IQR)*	4 (2–7)	4 (2–8)	0.066^4^
**Htc (%), median (IQR)**	47 (38–63)	50.9 (21–58)	0.177^2^
**Hb (g/dL), mean ± SD**	15.6 ± 2.5	16.6 ± 2.4	0.187^4^
**WBC (103** **μL), median (IQR)**	12145 (4690–32,580)	14310 (7480–19,310)	0.503^2^
**Platelets (103** **μL), median (IQR)**	323.5 (204–484)	295 (152–368)	**0.032** 2
**pH (1st hour), median (IQR)**	7.32 (7.23–7.48)	7.27 (7.16–7.41)	0.158^2^
**PCO_2_** **(mmHg), median (IQR)**	36 (20–52)	39.5 (29–52)	0.347^2^
**NT-proBNP (pg/mL), median (IQR)**	1779.5 (359–11,639)	2200 (632–26,594)	0.23^0^2
**24^th^** **hour of life**			
**pH, median (IQR)**	7.41 (7.20–7.59)	7.38 (7.19–7.54)	0.230^2^
**PCO_2_** **(mmHg), median (IQR)**	30 (16–123)	33.5 (26–44)	0.068^2^
**Procalcitonin (ng/mL), median (IQR)**	8.2 (0.1–40.2)	27.4 (0.2–69)	**0.004** 2
**CRP (mg/L), median (IQR)**	1.07 (0–20)	0.75 (0.01–4)	0.094^2^
**NT-proBNP (pg/mL), median (IQR)**	3308 (69–19,746)	7263.5 (1643–35,000)	**0.00**^4^2

1 Pearson chi-square test,

2 Mann–Whitney U test,

3 Fisher’s exact test,

4 Student’s *t*-test.

SBP: Systolic blood pressure; DBP: Diastolic blood pressure; TTN: Transient tachypnea of the newborn; NP: Neonatal pneumonia; Htc: Hematocrit; Hb: Hemoglobin; WBC: White blood cell; CRP: C-reactive protein.

* Silverman scores were calculated at 6th hour of life.

**Table 4:** Comparison of the NT-proBNP values, antibiotics, respiratory support data, and NICU duration between the low-score group and the high-score group.

	Groups according to LUS scores		
	Low-score group (n = 35)	High-score group (n = 23)	p
**NT-proBNP (pg/mL) 1^st^** **hour of hospitalization**	1870 (547–26,594)	1219 (359–21,300)	0.298^2^
**NT-proBNP (pg/mL) 24^th^** **hour of life**	3500 (570–15,948)	6320 (69–35,000)	**0.044** 2
**Antibiotics, n (%)**	19 (54)	17 (73)	0.132^1^
**PPV in the DR, n (%)**	8 (22)	4 (17)	0.746^3^
**Oxygen duration (hours)**	12 (6–140)	48 (4–168)	**0.006** 2
**NIV duration (hours)**	12 (6–120)	48 (4–144)	**0.008** 2
**Mechanical ventilation, n (%)**	1 (2)	4 (17)	0.075^3^
**NICU stay (days)**	4 (2–12)	9 (2–18)	**0.004** 2

1 Pearson chi-square test,

2 Mann–Whitney U test,

3 Fisher’s exact test.

TTN: Transient tachypnea of the newborn; NP: Neonatal pneumonia; PPV: Positive pressure ventilation; DR: Delivery room; NIV: Noninvasive ventilation; NICU: Neonatal intensive care unit.
